# Preparation and Anticorrosive Performance of Waterborne Epoxy Resin Composite Coating with Amino-Modified Graphene Oxide

**DOI:** 10.3390/polym15010027

**Published:** 2022-12-21

**Authors:** Ding Nan, Xin Li, Dongsheng Li, Qiong Liu, Biao Wang, Xin Gao, Ting Ma, Na He, Yu Xu, Junhui Dong

**Affiliations:** 1 Inner Mongolia Enterprise Key Laboratory of High Voltage and Insulation Technology, Inner Mongolia Power Research Institute Branch, Inner Mongolia Power (Group) Co., Ltd., Hohhot 010020, China; 2 College of Chemistry and Chemical Engineering, Inner Mongolia University, Hohhot 010021, China; 3 Inner Mongolia Key Laboratory of Graphite and Graphene for Energy Storage and Coating, School of Materials Science and Engineering, Inner Mongolia University of Technology, Hohhot 010051, China; 4 Inner Mongolia Institute of Metrology and Testing, Hohhot 010050, China

**Keywords:** 2,5-diaminobenzenesulfonic, modified graphene oxide, waterborne epoxy resin, corrosion resistance

## Abstract

A waterborne epoxy coating with superior corrosion resistance was developed by using a novel amino-functionalized graphene oxide (GO) that was modified by 2,5-diaminobenzenesulfonic acid. A battery of characterization methods, such as Fourier transform infrared spectroscopy (FT-IR), Raman spectra, X-ray diffraction (XRD), scanning electron microscopy (SEM), and transmission electron microscopy (TEM), was used to prove that DGO was successfully prepared by grafting the amino of 2,5-diaminobenzenesulfonic on GO. The results indicated that the surface of DGO became rougher than GO, but a complete sheet structure was still maintained after modification; the optimal modified GO could be achieved when the mass ratio of 2,5-diaminobenzenesulfonic acid and GO was 5:1. The electrochemical impedance spectroscopy (EIS) tests indicated that the impedance at 0.01 Hz of a coating with 0.2 wt.% DGO still remained at a relatively high value after immersion for 48 h in 3.5 wt.% NaCl, which was about one order higher than a pure waterborne epoxy resin coating, and the corrosion current density decreased from 3.76 × 10^−11^ A/cm^2^ to 3.62 × 10^−12^ A/cm^2^. The dry adhesion and wet adhesion increased to 1.90 and 1.22 MPa, respectively, and the adhesion loss decreased from 53% to 36%. These interesting features could make waterborne epoxy coatings a promising anticorrosion coating for metal in long-term protection.

## 1. Introduction

The corrosion of metal materials has attracted widespread attention due to huge economic losses and calamitous accidents. Nowadays, with the increasing application of batteries used in humid working environments, for instance, offshore wind energy harvesting, water infiltration through cracks in the outer metal packing, and even catastrophic accidents caused by corrosion cannot be ignored. Many measures, including cathode protection technology [1,2,3], the design of novel anticorrosive materials [4,5], and multifunctional anticorrosion coatings [6,7] have been used in metal materials. Among these strategies, multifunctional anticorrosion coatings have been widely used owing to their wide selectivity and applicability, excellent physical barrier performance, stable chemical properties, and low-cost [6,8,9]. However, organic solvent-borne coatings will cause environmental pollution, and large amounts of volatile organic compounds (VOCs) will be released during the curing process [10,11]. Therefore, traditional solvent-borne coatings are gradually being replaced by waterborne coatings, such as waterborne epoxy resins.

Waterborne epoxy resin coatings can be penetrated easily by molecules and corrosive ions, which cause the premature failure of the coating and the serious corrosion of the metal matrix; thus, further applications could be limited in many fields [12]. In order to enhance the physical barrier performance and corrosion resistance of waterborne epoxy resin coatings, Liu et al. [13] designed a two-step synthesizing process to prepare epoxy/acrylic composite waterborne resin-grafted (EA resin) copolymers for waterborne coatings. The degree of cross-linking of the coatings was enhanced by a moderate increase in the resin molecular weights (<8000 Da) and the carboxyl contents (<27 wt.%). Although an effective barrier effect of composite matrixes can be provided by longer epoxy-octanoic hydrophobic chains, the corrosion resistance of waterborne resins still needs to be further improved.

Due to superior impermeability and high specific surface area, graphene, a kind of unique two-dimensional layered material, can be used as excellent barrier reinforcement for protective coatings [14,15,16]. Liu et al. [17] used graphene as an inhibitor in waterborne epoxy coatings, and the corrosion protection performance of composite coatings with 0.5 wt.% graphene was significantly improved compared to pure epoxy coating. Actually, the dispersion of graphene is of crucial importance toward its performance and potential applications in waterborne coatings [18,19,20]. Therefore, GO with better dispersion has been more widely used in waterborne epoxy resin coatings. For instance, Xiao et al. [21] prepared a polyaniline-graphene oxide (PAGO) composite with superior dispersibility in zinc-based waterborne epoxy-acrylic coatings using an in-situ polymerization method. The cathodic protection and barrier performance of the coatings was enhanced by a small amount of PAGO. However, the particle size, concentration, and dispersion of zinc powder will decrease the corrosion-protective properties of coatings. In recent years, considering strong chemical bonds between epoxy and amino groups, some researchers used amino covalent bonds to enhance the dispersion of GO in solvent-based epoxy resin. Ramezanzadeh et al. [22] synthesized an amino-functionalized graphene oxide (FGO), and the corrosion resistance of the epoxy coating with FGO was significantly enhanced through the improvement of the dispersion of GO as well as barrier properties. Guo et al. [23] prepared aniline trimer-modified GO (ATGO) composites at different temperatures. The results indicated that the corrosion protection performance of the epoxy resin was significantly increased by ATGO, and the optimal reaction temperature for ATGO preparation was determined to be 95 °C. Therefore, the superior performance of amino-functionalized GO has been demonstrated in solvent-based epoxy resin anticorrosive coatings. However, research on amino-modified GO in the field of waterborne coatings is still lacking. Furthermore, the representative application of amino-functionalized graphene oxide still needs to be further investigated and confirmed.

In this work, we report a simple, scalable, and inexpensive approach for the preparation of a novel amino-functionalized GO that is modified by 2,5-diaminobenzenesulfonic acid (DGO), and we use it as a functional filler for a waterborne epoxy resin coating to improve its anticorrosive performance. Moreover, the optimal addition amount of DGO and the corrosion-protective properties of the hybrid coating were investigated.

## 2. Experimental

### 2.1. Materials

Graphite was purchased from Dasheng Graphite New Materials Co., Ltd., (Ulanqab, China), and 2,5-diaminobenzenesulfonic acid was purchased from Shanghai Macklin Biochemical Co., Ltd. Waterborne epoxy resin emulsion (component A) and waterborne epoxy curing agents (component B) were purchased from Shanghai Jiuyou Chemical Technology Co., Ltd. (Shanghai, China). All reagents were used as received without further purification. Q235 steel plates were selected for the salt spray corrosion test; they were polished with 400, 800, and 1200 SiC abrasive paper, then rinsed with distilled water, and, finally, dried in air.

### 2.2. Synthesis of GO and DGO

The modified Hummers method was adopted to prepare graphene oxide (GO). Firstly, graphite was added to a mixture of H_2_SO_4_ and H_3_PO_4_. Subsequently, KMnO_4_ was added slowly to an ice bath and stirred in a water bath at 60 °C for 8 h. After the reaction was completed, H_2_O_2_ was added slowly to remove excess KMnO_4_, and residual acids were removed using DI water several times. GO was obtained after filtration and drying.

After that, the GO and 2,5-diaminobenzenesulfonic acid were dispersed in 50 and 20 mL ultrapure water, respectively, with vigorous stirring for 1 h at room temperature to disperse thoroughly. Subsequently, the GO aqueous solution was mixed with a 2,5-diaminobenzenesulfonic acid aqueous solution in a mass ratio of 5:0.5, 5:1, and 5:1.5, which are denoted as DGO05, DGO10, and DGO15. Additionally, vigorous stirring for 40 min at 80 °C was performed to investigate the effect of the weight percent (wt.%) of 2,5-diaminobenzenesulfonic acid on the modification of GO. The final products were placed in a vacuum oven and dried overnight at 60 °C.

### 2.3. Preparation of Waterborne Epoxy Resin/DGO Coatings

**Figure 1:**
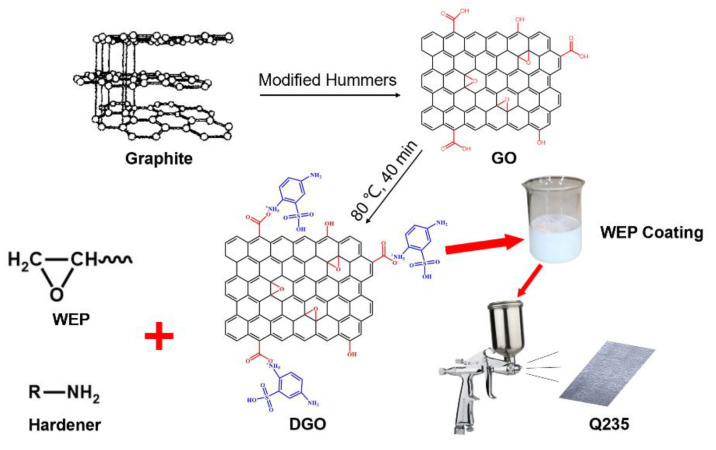
The synthesis scheme of DGO and waterborne epoxy resin coatings.

The DGO10 was adopted to prepare waterborne epoxy resin/DGO coatings. Different qualities of DGO10 were added to ultrapure water and sonicated for 1 h. The waterborne epoxy resin emulsion and the waterborne epoxy curing agents were then mixed and stirred in a mass ratio of 2:1. Subsequently, the waterborne epoxy resin coating was mixed with DGO aqueous solution and sonicated for 30 min. Finally, the waterborne epoxy resin/DGO coatings were obtained and then sprayed on the surface of pre-treated Q235 steel plates. The preparation process of the DGO and waterborne epoxy resin/DGO coatings is schematically illustrated in Figure 1. The thickness of coatings was controlled within the range of 30 ± 10 μm. To investigate the effect of DGO on the anticorrosion properties of coatings, DGO10 samples with a mass ratio of 0.05%, 0.1%, 0.2%, and 0.3% (vs. the mass of epoxy) were added to the coatings and denoted as DGO10/WEP-05, DGO10/WEP-10, DGO10/WEP-20 and DGO10/WEP-30, respectively; the coating without added DGO10 was named WEP.

### 2.4. Characterizations

Fourier transform infrared (FT-IR) spectroscopy of GO and DGO were performed using an FT-IR spectrometer (Bruker TENSOR II, Karlsruhe, Germany). All spectra were carried out between 4000 and 400 cm^−1^. The X-ray diffraction (XRD) patterns were obtained using an X-ray diffractometer (D/MAX-2500/PC). The radiation source was Cu *K_α_*. Raman spectra were obtained on a confocal microscopic Raman spectrometer (Horiba IHR320, Kyoto, Japan) with 532 nm laser light irradiation from 100 to 4000 cm^−1^. The morphology of GO and DGO was observed using scanning electron microscopy (SEM, FEIXL30 ESEM FEG, USA) and transmission electron microscopy (TEM, JEOL JEM2100, Tokyo, Japan). The anticorrosion properties of the waterborne epoxy resin coating samples were evaluated by electrochemical impedance spectroscopy (EIS) using an electrochemical workstation (Princeton PMC-1000A, Irvine, CA, USA) and a salt spray test. The EIS analysis was carried out in a conventional three-electrode cell, where a saturated calomel electrode was used as the reference electrode, platinum as the counter electrode, and steel samples coated with EP waterborne epoxy resin coatings as the working electrode. Additionally, the frequency was in the range of 100 kHz to 10 mHz, the amplitude was 20 mV, and the scan rate was 1 mV/s. Salt spray exposure was performed for 300 h according to ASTM B117.

## 3. Results and Discussion

### 3.1. Characterization of DGO

**Figure 2:**
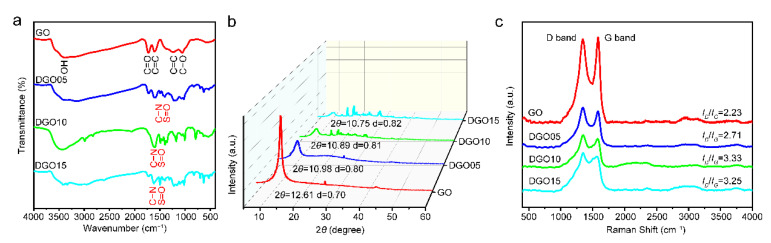
FT-IR spectra (**a**), XRD spectra (**b**) and Raman spectra (**c**) of GO, DGO05, DGO10 and DGO15.

FT-IR analysis was performed to investigate the effect of the weight percent (wt.%) of 2,5-diaminobenzenesulfonic acid on the modification of GO. Figure 2a shows the FT-IR spectra for GO, DGO05, DGO10, and DGO15 nanosheets. According to Figure 2a, the main peaks related to the GO structure at 3379, 1728, 1632, and 1067 cm^−1^ were observed, corresponding to −OH, C=O, C=C, and C−O−C, respectively. A dramatic decrease in the intensity of C=O at 1728 cm^−1^ and the advent of C−N at 1500 cm^−1^ suggest the chemical reactions between carboxyl and amino groups. Additionally, the occurrence of S=O at 1400 cm^−1^ proves the covalent bonds between 2,5-diaminobenzenesulfonic acid and GO. The cluttered peaks in the FT-IR spectrum of DGO15 are attributed to the excessive 2,5-diaminobenzenesulfonic acid. Therefore, the optimal modification can be achieved when the mass ratio of 2,5-diaminobenzenesulfonic acid and GO is 5:1, as shown in the FT-IR spectrum of DGO10.

XRD patterns were further presented to illustrate the modification effect, and the interlayer spacing of GO, DGO05, DGO10, and DGO15 are shown in Figure 2b. The diffraction peak of (002) at 2θ = 12.61° illustrated that the interlayer spacing of GO was 0.7 nm. However, the (002) peaks of DGO05, DGO10, and DGO15 shifted from 12.61° to 10.98°, 10.89°, and 10.75°, indicating the interlayer spacing of DGO05, DGO10, and DGO15 was 0.80, 0.81, and 0.82 nm, respectively. The resulting larger interlayer distance of DGO05, DGO10, and DGO15 can be ascribed to the wrinkling and stack of functional groups of modified GO caused by the successful modification chemical reaction between GO and 2,5-diaminobenzenesulfonic acid.

The disorder and defect structure of GO, DGO05, DGO10, and DGO15 were characterized by Raman spectroscopy, as shown in Figure 2c. The D-band of GO, shown at 1347 cm^−1^, corresponded to the defects, edge effects, and disorder platelets. The G-band shown at 1596 cm^−1^ corresponded to the first-order scattering of the *E_2g_* vibration mode and the in-plane vibration of ordered sp^2^-bonded carbon atoms [24,25,26]. A noticeable intensity change in the Raman spectrums of DGO05, DGO10, and DGO15 can be observed in Figure 2c, but there is no band shift compared to GO. The intensity ratio of the D-band to G-band (*I_D_*/*I_G_*) increased from 2.23 for GO to 2.71 for DGO05, 3.33 for DGO10, and 3.25 for DGO15, respectively. The high degree of reduction and disorder of DGO implies that reacting sites located on the GO platelets are attacked by the 2,5-diaminobenzenesulfonic acid [24]. Moreover, these results indicate the further distortion of bonds and the extensive destruction of symmetry due to a reduction in the size of in-plane sp^2^ domains resulting from the extra grafting with 2,5-diaminobenzenesulfonic acid.

**Figure 3:**
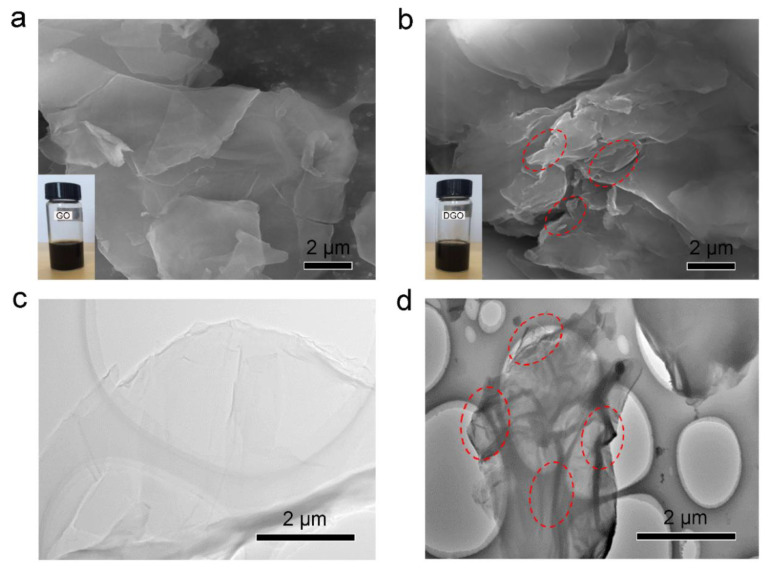
SEM images of GO (**a**) and DGO (**b**) (the insets were optical photographs of GO and DGO after being dispersed in water for 100 h), and TEM images of GO (**c**) and DGO (**d**).

In summary, combined with FT-IR, XRD, and Raman analysis, it is concluded that optimal modified GO can be achieved when the mass ratio of 2,5-diaminobenzenesulfonic acid with GO is 5:1, which be used as a functional filler in the subsequent preparation of waterborne epoxy resin coatings. The water dispersion of GO and DGO10 was tested in water for 100 h. Additionally, the SEM and TEM images of GO and DGO10 are shown in Figure 3. The smooth surface of GO can be observed clearly in Figure 3a,c, which indicates that GO was successfully synthesized using a modified Hummers method. The surface of DGO10 became rougher than GO but still maintained a complete sheet structure (indicated with red circles in Figure 3), as shown in Figure 3b,d. Further, the increased interlayer spacing of segregated GO nanosheets, which is confirmed by XRD analysis in Figure 2b, can be observed by comparing the SEM and TEM images of GO and DGO1.

### 3.2. Characterization of WEP/DGO Composites

The effect of the weight percent of DGO10 on the properties of waterborne epoxy resin coatings was then investigated by using 0.05, 0.1, 0.2, and 0.3 wt.% DGO10, and correspondingly, the waterborne epoxy resin coatings were denoted as DGO10/WEP-05, DGO10/WEP-10, DGO10/WEP-20, and DGO10/WEP-30, respectively; the waterborne epoxy resin coating without DGO was named WEP.

**Figure 4:**
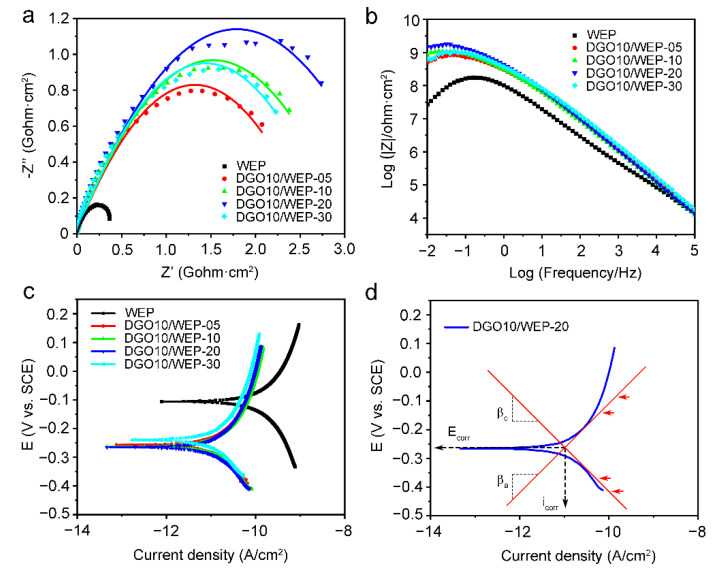
Nyquist diagrams (**a**), Bode diagrams (**b**), and potentiodynamic polarization curves (**c**) of waterborne epoxy resin coatings after immersion in 3.5 wt.% NaCl solution for 48 h. Tafel extrapolation on the polarization plot of the DGO10/WEP-20 sample for the determination of the corrosion current and Tafel slopes (**d**).

The shielding and corrosion protection properties of waterborne epoxy resin coatings with DGO for different additions immersed in 3.5 wt.% NaCl solution for 48 h were investigated using EIS and polarization curves, as shown in Figure 4. The radius of capacitive arcs increased in a certain range with the addition of DGO10; when the addition of DGO10 was 0.2 wt.%, the radius of DGO10/WEP-20 was the largest (Figure 4a), indicating the optimal corrosion-protective properties. Moreover, the anticorrosion properties of coatings can be characterized by impedance modulus (|Z|_0_._01Hz_) [7]. The |Z|_0_._01Hz_ of DGO10/WEP-20 was about one order higher than that of WEP, which demonstrates the superior barrier characteristics and corrosion-protective properties of the DGO10/WEP-20 sample. Notably, only one relaxation time can be seen in the Bode diagram of all coating samples after 48 h immersion in the range of 100 kHz to 10 mHz, as shown in Figure 4b, which indicates that the corrosive medium penetrated the coating but did not arrive at the coating/metal interface and that the electrochemical events in this work are mainly under the control of the ionic resistance (barrier effect) of the waterborne epoxy resin coating [23].

The corrosion current density (I_corr_) of WEP is 3.76 × 10^−11^ A/cm^2^, demonstrating it is susceptible to corrosion. However, all the corrosion potentials (E_corr_) of waterborne epoxy resin coatings with DGO shift to more negative potentials; I_corr_ significantly decreases, as shown in Figure 4c, and the I_corr_ of DGO10/WEP-20 decreased to 3.62 × 10^−12^ A/cm^2^ by one order of magnitude, confirming the promotion of DGO10 on the superior corrosion protection properties of waterborne epoxy resin coatings. Besides, both anodic and cathodic Tafel slopes are changed by the incorporation of DGO10, indicating that the anticorrosion properties of waterborne epoxy resin coatings were changed via the DGO10 addition. Accordingly, the shielding properties and corrosion protection properties of waterborne epoxy resin coatings were enhanced by DGO10.

**Figure 5:**
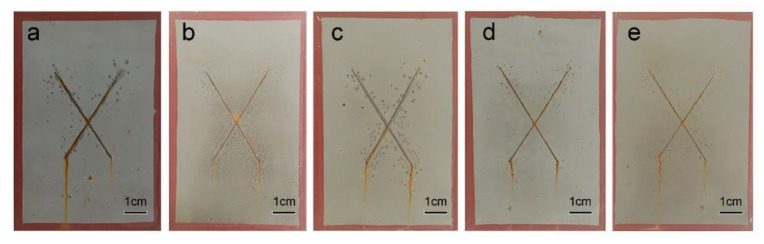
Waterborne epoxy resin coatings exposed to the salt spray test for 300 h. (**a**) WEP; (**b**) DGO10/WEP-05; (**c**) DGO10/WEP-10; (**d**) DGO10/WEP-20; (**e**) DGO10/WEP-30.

The anticorrosion properties of waterborne epoxy resin coatings were investigated using a salt spray test for 300 h, according to ASTM B117 standard, as shown in Figure 5. Significant corrosion products and lots of bubbles produced around scribes and beneath the coating can be observed in Figure 5a, which indicates poor corrosion resistance and the shielding effect of WEP. Corrosion products around scribes were significantly reduced when 0.05 wt.% of DGO10 was added into DGO10/WEP-05, but there were still lots of bubbles, as shown in Figure 5b. When the amount of DGO10 was 0.1 and 0.2 wt.%, the number of corrosion products and bubbles on the surface of DGO10/WEP-10 and DGO10/WEP-20 were gradually reduced, as shown in Figure 5c–d. It is worth noting that there are almost no corrosion products or bubbles on the surface of DGO10/WEP-20, which demonstrates the optimal corrosion protection and shielding effect, as shown in Figure 5d. However, with the continuous addition of DGO10, corrosion products and bubbles gradually increase on the surface of DGO10/WEP-30; these are attributed to the uneven distribution of redundant DGO10.

**Figure 6:**
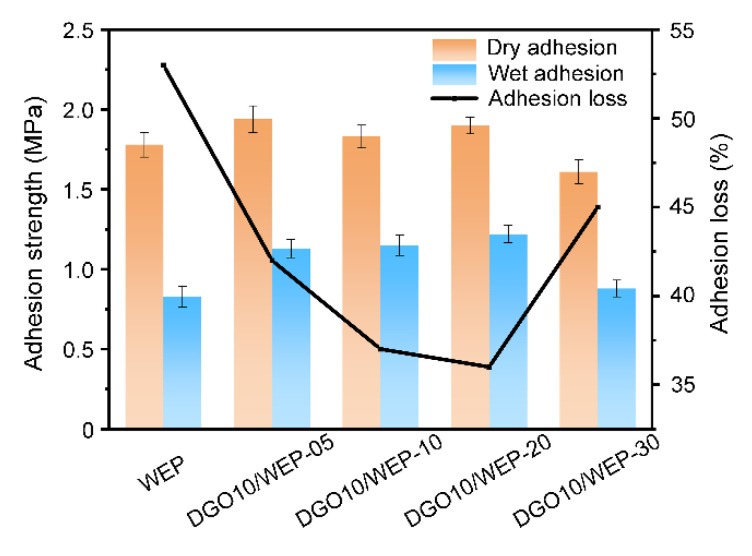
Pull-off adhesion results of waterborne epoxy resin coatings.

At the initial stage of the corrosion process of the metal matrix, OH^−^ is generated at the interface between the coating and the metal (2H_2_O + O_2_ + 4e^−^ → 4OH^−^), leading to a local pH increase on the metal surface, which aggravates hydrolysis and causes a decrease in the adhesion of the coating. In addition, the corrosion products at the interface continue to accumulate as the corrosion degree gradually increases, which will further reduce the adhesion of the waterborne epoxy resin coating [23]. In this work, the dry adhesion and wet adhesion (immersion in 3.5% NaCl for 7 days) of waterborne epoxy resin coatings were investigated by using a pull-off adhesion tester, and the results are shown in Figure 6.

It can be seen from Figure 6 that the dry adhesion and wet adhesion of WEP are 1.78 and 0.83 MPa, respectively. For the coatings with 2 wt.% DGO10, both dry adhesion and wet adhesion increased significantly. The dry adhesion of DGO10/WEP-20 was 1.90 MPa, which is 1.07 times that of WEP. Additionally, the wet adhesion was 1.22 MPa, which is 1.47 times that of WEP. Dramatically, the adhesion loss of DGO10/WEP-20 decreased from 53% to 36%, which indicates that the barrier protection performance was significantly improved by 2 wt.% DGO10.

### 3.3. Discussion

**Figure 7:**
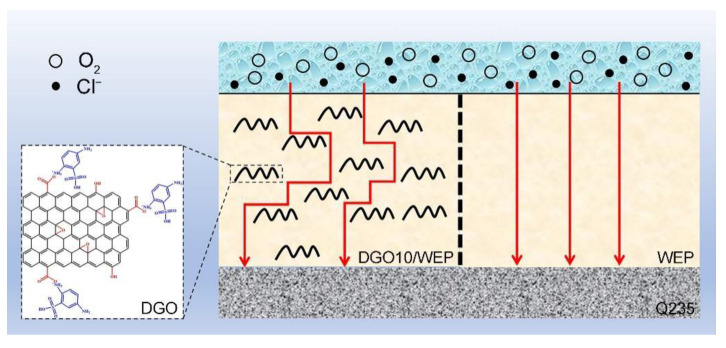
Schematic representation of the corrosion protection mechanism by DGO on Q235 steel substrates.

Corrosion of the metal matrix under the coating is caused by the redox reaction of the corrosive medium (H_2_O, O_2,_ and Cl^−^), which penetrates the coating through cracks and micropores to reach the interface of the coating and metal [27]. It is worth noting that H_2_O and O_2_ are necessary factors for the redox reaction. Therefore, the redox reaction on the surface of the metal matrix can be effectively prevented by preventing H_2_O and O_2_ from penetrating the coating to reach the metal surface. In our work, a “maze effect” was formed in the waterborne epoxy resin coating using DGO as a functional filler, as shown in Figure 7, which effectively extended the pathways for the corrosive medium to penetrate the coating and significantly improved the shielding and corrosion-protective properties. Moreover, the number of defects and micropores in the waterborne epoxy resin coating decreased significantly, and the local anode and cathode directions on the surface of the metal matrix were blocked effectively. Therefore, the shielding and corrosion-protective properties of DGO10/WEP were improved.

Furthermore, as shown in Figure 4a,b, Figure 5 and Figure 6e, the corrosion-protective properties were reduced by excessive DGO due to the aggregation of DGO in coatings. In this work, the optimal corrosion-protective property was obtained in DGO10/WEP-20, which used 0.2 wt.% DGO, and it was decreased in the order of DGO10/WEP-20, DGO10/WEP-30, DGO10/WEP-10, DGO10/WEP-05, and WEP. Consequently, the optimal chemical bonding and corrosion-protective property was obtained by the appropriate amount of DGO (0.2 wt.%).

## 4. Conclusions

In conclusion, a waterborne epoxy coating with superior anticorrosion properties was synthesized by a facile, scalable, and inexpensive approach for introducing the multifunctional composite filler DGO with amino-modified GO by 2,5-diaminobenzenesulfonic acid. The optimal modified GO (DGO10) was achieved when the mass ratio of 2,5-diaminobenzenesulfonic acid and GO was 5:1. The surface of DGO10 became rougher than pure GO but still maintained a complete sheet structure. The interlayer spacing of DGO10 increased from 0.7 to 0.81 nm, *I_D_/I_G_* increased from 2.23 to 3.33, and the surface became rougher with a complete sheet structure. The optimal corrosion protection property of waterborne epoxy resin coatings was achieved in DGO10/WEP-20, which used 0.2 wt.% DGO. Electrochemical impedance spectroscopy (EIS) tests revealed that the impedance at 0.01 Hz of DGO10/WEP-20 still remained at a relatively high value after 48 h of immersion in 3.5 wt.% NaCl, which was about one order higher than that of WEP, and the corrosion current density decreased from 3.76 × 10^−11^ A/cm^2^ to 3.62 × 10^−12^ A/cm^2^. Additionally, there were almost no corrosion products or bubbles on the surface of DGO10/WEP-20 after the salt spray test for 300 h. The dry adhesion and wet adhesion of DGO10/WEP-20 increased from 1.78 and 0.83 MPa to 1.90 and 1.22 MPa, respectively, and the adhesion loss decreased from 53% to 36%. These interesting features could make waterborne epoxy coatings a promising anticorrosion coating for metals in long-term anticorrosive protection.

## Data Availability

Not applicable.
